# Triage misalignment and early neonatal death in regionalized perinatal care

**DOI:** 10.3389/frph.2026.1944917

**Published:** 2026-10-13

**Authors:** Md Asif Khan Sharon, Sandra D. Eksioglu, Cari Anne Bogulski, Austin Porter, Enrique Gomez-Pomar

**Affiliations:** 1 Department of Industrial Engineering, University of Arkansas, Fayetteville, AR, United States; 2 Department of Biomedical Informatics, University of Arkansas for Medical Sciences, NW Campus, Fayetteville, AR, United States; 3 Department of Health Policy and Management, University of Arkansas for Medical Sciences, Little Rock, AR, United States; 4 Institute for Community Health Innovation, University of Arkansas for Medical Sciences, Springdale, AR, United States; 5 Department of Pediatrics, University of Arkansas for Medical Sciences, Little Rock, AR, United States

**Keywords:** early neonatal mortality, maternal care access, neonatal care levels, obstetric unit closures, perinatal regionalization of care, risk-appropriate care, triage misalignment

## Abstract

**Background:**

Regionalized perinatal systems aim to align maternal–infant risk with facility capability, yet most evidence focuses on extremely preterm and very low birth weight infants and evaluates delivery hospital or NICU outcomes in isolation. How triage alignment across the full perinatal care continuum relates to early neonatal mortality, including in lower-risk infants and in settings with declining obstetric capacity, is not well understood.

**Methods:**

We conducted a population-based retrospective cohort study of 428,717 live births (2013–2024) in Arkansas, including 1,511 early neonatal deaths (0–6 days). Triage alignment at birth, NICU admission, and interfacility transfer was classified as risk-appropriate, under-triaged, or over-triaged based on concordance between required and actual facility level within a regionalized system. Matched case–control analyses with conditional logistic regression used a sequential modeling strategy distinguishing pre-delivery confounders (gestational age, birth weight) from post-delivery clinical indicators (Apgar score, ventilation, seizures, congenital anomalies), with a nested sensitivity analysis to assess mediation.

**Results:**

Under-triage at birth was associated with five-fold higher early neonatal mortality than risk-appropriate care (56.89 vs. 10.70 per 1,000; *P* < 0.001). After adjustment for gestational age and birth weight, under-triage remained the strongest independent predictor of early neonatal death (OR 5.85; 95% CI, 4.43–7.72), including among term and normal-birth-weight infants. Distance greater than 30 miles to the delivery facility was independently associated with mortality (OR 2.90; 95% CI, 2.21–3.81), and limited prenatal care remained independently associated with mortality (0 visits: OR 1.51; 95% CI, 1.01–2.24; 1–3 visits: OR 1.78; 95% CI, 1.22–2.59). Localized increases in early neonatal mortality were observed following obstetric unit closures despite relatively stable statewide trends. Black/African American infants had nearly double the early neonatal mortality rate of White infants (5.49 vs. 2.90 per 1,000), with lower prenatal care utilization.

**Conclusions:**

Within a regionalized perinatal system, under-triage at birth and inadequate prenatal care are major determinants of early neonatal death across the risk spectrum. Prenatal risk identification, sufficient engagement with prenatal care, triage alignment at birth, geographic access, and coordinated referral should be treated as core system-level targets for reducing preventable early neonatal mortality.

## 1 Introduction

The early neonatal period, the first seven days of life, remains the highest-risk window for infant mortality worldwide; an estimated three-quarters of neonatal deaths occur in this interval, with more than half in the first 48 h of life (1–3). In the United States, early neonatal mortality is a persistent contributor to infant mortality, driven largely by complications of preterm birth, intrapartum-related events, and congenital anomalies (1, 3). Because the causes and timing of these deaths are tightly coupled to conditions around delivery and immediate postnatal care, survival depends critically on alignment between an infant's clinical needs and the capability of the facilities where care is delivered.

Regionalized perinatal systems were developed to promote “risk-appropriate care” by organizing maternal and neonatal services across graded levels of facility capability and concentrating high-risk pregnancies in centers equipped to provide advanced care (4, 5). The American Academy of Pediatrics (AAP) codified levels of neonatal care (I–IV), specifying staffing, equipment, and clinical capabilities for each level (5). A substantial body of evidence demonstrates that very low birth weight and extremely preterm infants have better outcomes when born in high-level, high-volume centers rather than lower-level hospitals. Meta-analysis by Lasswell et al. showed substantially higher mortality for very low birth weight infants born outside Level III centers, with even larger effects among extremely low birth weight infants, and subsequent work by Jensen and Lorch linked NICU level and volume to morbidity and mortality (6, 7). Studies of neonatal transfer reinforce the importance of birth location: in a large English cohort, Helenius et al. found that extremely preterm infants born in non-tertiary hospitals and transferred within 48 h had higher odds of severe brain injury than infants born in tertiary centers, and Mori et al. reported that prolonged transport duration was associated with increased neonatal mortality (8, 9).

Despite this strong evidence base, important gaps remain. Most studies evaluate delivery hospital or NICU outcomes in isolation and focus primarily on extremely preterm and very low birth weight infants; few examine how triage alignment across multiple stages of the perinatal care continuum (prenatal risk identification, delivery location, NICU admission, and interfacility transfer) relates to early neonatal mortality at the population level, including among infants traditionally considered lower risk. Prenatal care is an important upstream determinant of early neonatal survival because it supports risk identification, referral, and planned delivery in appropriately resourced facilities, whereas limited prenatal care may delay recognition of risk until the intrapartum or immediate postnatal period (10, 11). The processes that determine whether risk-appropriate care is achieved—risk identification, referral pathways, stabilization capacity, and transfer coordination—have received less empirical attention than outcomes associated with specific facility levels. In parallel, national trends suggest de-regionalization: lower-level NICUs have proliferated in urban areas, and the proportion of high-risk infants delivered at high-volume tertiary centers has declined (12, 13). State neonatal risk-appropriate care policies are inconsistent, and many do not fully align with AAP standards; Kroelinger and colleagues reported that only about half of states had a neonatal risk-appropriate policy and that criteria for higher-level units were often incomplete (14, 15). CDC's LOCATe assessments further showed frequent discrepancies between facilities’ self-reported neonatal care levels and independent classification (14, 16).

These challenges are amplified in settings experiencing reductions in obstetric capacity. The proportion of rural U.S. counties with hospital-based obstetric services has declined over the past decade, and the availability of higher-level neonatal care in rural birth hospitals remains far lower than in urban hospitals (17, 18). Loss of rural obstetric services has been associated with increases in out-of-hospital births, deliveries in hospitals without obstetric units, and preterm births (17, 19). However, the relationship between obstetric unit closures and neonatal outcomes is not uniformly negative: in an urban setting, Wesselius et al. reported decreased perinatal mortality after closure of a community hospital obstetric unit, attributed to centralization and increased volume (20). A systematic review by Malouf et al. and recent work by Durrance et al. highlight that the impact of closures and travel time depends on availability of alternative facilities, referral networks, and broader system context (21, 22).

Within this context, we used statewide linked birth and death data from Arkansas to evaluate early neonatal mortality across the full perinatal care continuum—birth, NICU admission, and interfacility transfer—in a regionalized system undergoing obstetric unit closures. We conceptualized triage alignment as the concordance between an infant's required level of care and the level of the treating facility at each stage and classified care as risk-appropriate, under-triaged, or over-triaged (5). As a complement to our prior Arkansas analysis of risk-appropriate care and regionalization patterns, this study had several objectives (23). We examined the association between under-triage at birth and early neonatal death across the risk spectrum, including among term and normal-birth-weight infants, and assessed this association using a sequential modeling framework that distinguished pre-delivery confounders from post-delivery mediators. We also evaluated whether prenatal care utilization, distance to delivery facility, payment source, and obstetric unit closures were associated with triage alignment and early neonatal mortality within a regionalized perinatal system. More broadly, we applied an integrated analytical framework to examine how clinical and system-level factors jointly shape early neonatal survival.

## 2 Methods

### 2.1 Study design and data sources

We conducted a population-based retrospective cohort study of live births within a regionalized perinatal system using linked vital records and administrative data from 2013 to 2024. Birth records were obtained from the Arkansas All-Payer Claims Database, and infant death certificates from the Arkansas Department of Health, Division of Vital Records. Records were deterministically linked using a two-step strategy: primary linkage by birth certificate number followed by secondary linkage of unmatched birth records using a unique child identifier, infant sex, and maternal county of residence. When duplicate links occurred, primary birth-certificate matches were prioritized, and records were deduplicated to one linked death record per birth certificate before merging death indicators back to the birth cohort. Causes of death were classified using International Classification of Diseases, Tenth Revision (ICD-10) codes, consistent with National Center for Health Statistics definitions (24). The University of Arkansas for Medical Sciences Institutional Review Board determined that the study was non-human-participants research (IRB# 298850).

### 2.2 Study population

The source birth file included 431,117 live births from 2013 to 2024. After restricting to births with valid Arkansas maternal home counties, the final study population comprised 428,717 live births across 75 counties, linked to 3,577 infant deaths, including 1,511 early neonatal deaths (0–6 days), 428 late neonatal deaths (7–27 days), and 1,180 postneonatal deaths (28–364 days). Analyses requiring facility-level, access, NICU admission, prenatal visits, or payment type were restricted to births from 2014 to 2024 (*n* = 390,298) due to incomplete data capture in 2013. For interfacility transfer analyses, we included only transfers to hospitals with known neonatal care levels; transfers to facilities without classifiable levels (“No NICU”) were excluded. Under-triaged transfers were rare (*n* = 30; two early neonatal deaths) and were excluded from inferential transfer-triage analyses, a conservative choice that likely biases associations toward the null. Because most prior regionalization studies have focused on extremely preterm and very low birth weight infants, we additionally stratified analyses by gestational age and birth-weight categories to assess whether associations between triage alignment and mortality extend to lower-risk neonatal populations (6, 7).

### 2.3 Exposure variables

The primary exposure was triage alignment, defined as concordance between an infant's required level of neonatal care and the level of the treating facility. For birth (delivery hospital), NICU admission, and interfacility transfer (receiving hospital), we classified care as risk-appropriate (facility level ≥ required level), under-triaged (facility level < required level), or over-triaged (facility level > required level). Facility levels (I–IV) were verified using the Arkansas Department of Health perinatal regionalization list and cross-referenced with the Arkansas Center for Health Improvement hospital roster and the U.S. Maternal Health Portal (25–27). Required level of care was assigned using gestational age, birth weight, and clinical indicators, including assisted ventilation, prolonged ventilation, seizures, surfactant use, major congenital anomalies, and 5 min Apgar score, mapped to AAP guidance on levels of neonatal care (5). Infants with major surgical anomalies were assigned Level IV care; very preterm (<32 weeks), very low birth weight (<1,500 g), or had high-acuity neonatal indicators, including prolonged ventilation, seizures, severe 5 min Apgar depression, or surfactant use were assigned Level III; Infants who were at least 32 weeks' gestation and at least 1,500 g and had specialty-care indicators, including gestational age <35 weeks, short assisted ventilation, or moderate 5 min Apgar depression were assigned Level II; and remaining uncomplicated births were assigned Level I.

Distance to the delivery facility was calculated as the straight-line geodesic distance in miles from the centroid of the maternal county of residence to the delivery hospital and dichotomized at >30 miles, consistent with prior literature on rural perinatal outcomes and the March of Dimes maternity care desert definition (2, 21). Prenatal care was assessed using two variables: (1) any documented prenatal care vs. none or unknown, and (2) number of prenatal visits categorized into bands (0, 1–3, 4–7, 8–10, ≥11). The binary measure combined “no recorded prenatal care” with “unknown prenatal care status” because both reflect absence of documented engagement with prenatal services; potential misclassification of unknown status is addressed in the Limitations. Additional exposures included gestational age (completed weeks), birth weight (grams), primary payment source (private insurance, Medicaid, self-pay, other, unknown), county-level maternity care access based on March of Dimes maternity care desert classifications, and obstetric unit closures (pre- and post-closure periods) identified from state reports (2).

### 2.4 Outcome measures

The primary outcome was early neonatal mortality, defined as death within 0–6 completed days of life, consistent with WHO definitions (3). Secondary outcomes included late neonatal mortality (7–27 days) and postneonatal mortality (28–364 days). Mortality rates were calculated per 1,000 live births with Wilson 95% confidence intervals.

### 2.5 Statistical analysis

We summarized mortality rates across clinical, demographic, and system-level factors. Group differences were evaluated using chi-square or Fisher exact tests, with Cramér's V as a measure of effect size, and continuous variables using Mann–Whitney *U* or Kruskal–Wallis tests with Dunn *post hoc* comparisons as appropriate. When multiple pairwise comparisons were conducted within an analysis (e.g., across birth-weight or gestational age strata), Bonferroni-corrected significance thresholds were applied and reported in the corresponding figures. Temporal trends in early, late, and postneonatal mortality were assessed using nonparametric tests across study years. Geographic patterns were examined using county-level aggregation and mapping of early neonatal mortality and maternity care access categories. For counties experiencing obstetric unit closures, we compared early neonatal mortality pre-closure (births from 2014 to the closure date) and post-closure (births from the closure date through 2024) using odds ratios and Fisher exact tests; given small numbers of post-closure events and lack of concurrent control counties, these analyses were considered hypothesis-generating (20, 21).

To evaluate the independent association between triage alignment and early neonatal mortality while respecting the temporal ordering of variables, we used matched case-control analyses with conditional logistic regression and specified three nested models. When standard conditional logistic regression did not converge cleanly or showed evidence of quasi-separation, Firth-penalized conditional logistic regression was used to reduce small-sample and rare-event bias (28, 29). Cases were early neonatal deaths, and controls were surviving infants matched on key demographic variables. Model 1 included system-level exposures (under-triage at birth, distance >30 miles, prenatal visit bands, Medicaid/self-pay vs. private insurance). Model 2, designated as the primary causal model, added gestational age and birth weight as pre-delivery confounders determined before the triage exposure and strongly predictive of both facility selection and mortality (6, 7). Model 3 additionally included post-delivery clinical indicators—5 min Apgar score categories, assisted ventilation, prolonged ventilation, seizures, congenital anomaly, and infant sex—to allow comparison with conventional fully adjusted models, recognizing that these variables may lie on the causal pathway between under-triage and death (30, 31). We interpreted Model 2 as the primary estimate of the causal association between under-triage and early neonatal mortality because it adjusts for the main pre-delivery clinical confounders while avoiding overadjustment for potential mediators (32–34). To visualize effect stability and attenuation, we conducted a nested sensitivity analysis in which odds ratios and 95% confidence intervals for shared covariates were plotted side-by-side across the three models. This analysis informed interpretation of whether attenuation in Model 3 reflects mediation through post-delivery clinical deterioration rather than resolution of confounding (35, 36).

As an exploratory complement to regression-based analyses, we fit an extreme gradient boosting (XGBoost) model to assess nonlinear associations and interactions among predictors of early neonatal mortality. The binary outcome was early neonatal death (0–6 days), and the feature set included biological, clinical, prenatal care, access, payer, and demographic variables used in the regression workflow. Predictive performance was evaluated using 5-fold cross-validation that preserved matched sets within folds and was summarized using the area under the receiver operating characteristic curve (AUC) and the Brier score. A final XGBoost model was fit on the full matched analytic sample to estimate SHAP (SHapley Additive exPlanations) values for global feature-importance ranking. Because this analysis was exploratory and prediction-oriented, SHAP values were interpreted as measures of predictive contribution rather than causal effects. For additional information, please see Supplementary Methods and Model-Based Analyses.

In *post hoc* sensitivity analyses prompted by the increase in early neonatal mortality in 2023–2024, we compared 2014–2019 with 2023–2024 with respect to gestational-age-specific mortality, recorded maternal and neonatal COVID-19, prenatal visit frequency, and birth setting. Two-sided Fisher exact tests compared the proportion of births resulting in early neonatal death between periods within strata. We additionally repeated the distance-to-delivery analysis after restricting the cohort to Arkansas residents to assess the robustness of findings to potential cross-state delivery patterns.

All data management, linkage quality assurance, and statistical analyses were performed in Python version 3.7.6 using standard scientific computing libraries (*pandas, NumPy, SciPy, and statsmodels)*. Statistical significance was defined as a two-sided *P* value < 0.05. Figures were generated using *Matplotlib* and *Seaborn*.

## 3 Results

### 3.1 Study population

The final study population included 428,717 live births from 2013 to 2024 across 75 Arkansas counties, linked to 3,577 infant deaths, including 1,511 early neonatal deaths (0–6 days), 428 late neonatal deaths (7–27 days), and 1,180 postneonatal deaths (28–364 days). Descriptive and demographic characteristics are presented in Table 1. Analyses involving facility-level, access, and payment variables were restricted to births from 2014 to 2024 (*n* = 390,298 births; 1,389 early neonatal deaths) due to incomplete data capture in 2013.

**Table 1:** Baseline characteristics of early neonatal deaths and matched surviving controls, Arkansas, 2014–2024.

Characteristic	Level	Early neonatal deaths, *n* (%) (*n* = 1,075)	Surviving matched controls, *n* (%) (*n* = 2,758)	*p* value
Maternal characteristics				
Maternal age, years	Median [IQR]	27.0 [23.0, 31.0]	27.0 [23.0, 32.0]	0.990
Maternal race	White	680 (63.3)	1,574 (57.1)	0.015
	Black	269 (25.0)	848 (30.7)	
	Asian	23 (2.1)	62 (2.2)	
	Native American	6 (0.6)	18 (0.7)	
	Pacific Islander	12 (1.1)	35 (1.3)	
	Unknown	85 (7.9)	221 (8.0)	
Maternal Hispanic origin	Non-Hispanic	939 (87.3)	2,395 (86.8)	0.708
	Hispanic	107 (10.0)	296 (10.7)	
	Unknown	29 (2.7)	67 (2.4)	
Parity	0	996 (92.7)	2,562 (92.9)	0.275
	1	79 (7.3)	187 (6.8)	
	2	0 (0.0)	7 (0.3)	
	≥3	0 (0.0)	2 (0.1)	
Infant clinical characteristics				
Gestational age, weeks	Median [IQR]	28.0 [22.0, 36.0]	30.0 [26.0, 36.0]	<0.001
Gestational age category	Extremely Preterm	522 (48.6)	1,178 (42.7)	0.005
	Very Preterm	121 (11.3)	338 (12.3)	
	Preterm	202 (18.8)	566 (20.5)	
	Term	224 (20.8)	671 (24.3)	
	Post Term	3 (0.3)	4 (0.1)	
	Unknown	3 (0.3)	1 (0.0)	
Birth weight, grams	Median [IQR]	1,077.0 [482.0, 2,251.5]	1,276.0 [770.0, 2,466.0]	<0.001
Birth weight category	ELBW	521 (48.5)	1,201 (43.5)	<0.001
	VLBW	130 (12.1)	333 (12.1)	
	LBW	201 (18.7)	598 (21.7)	
	Normal	191 (17.8)	569 (20.6)	
	Macrosomia	20 (1.9)	56 (2.0)	
	Unknown	12 (1.1)	1 (0.0)	
Prenatal care and payer				
Prenatal care status	Any prenatal care	836 (77.8)	2,260 (81.9)	0.004
	No/unknown prenatal care	239 (22.2)	498 (18.1)	
Prenatal visit count	Median [IQR]	5.0 [2.0, 9.0]	6.0 [4.0, 10.0]	<0.001
Payment source	Medicaid	473 (44.0)	1,227 (44.5)	0.914
	Private Insurance	516 (48.0)	1,331 (48.3)	
	Self-Pay	47 (4.4)	102 (3.7)	
	Other	11 (1.0)	27 (1.0)	
	Unknown	28 (2.6)	71 (2.6)	
Access and delivery characteristics				
County-level maternity care access	Full Access	768 (71.4)	2,031 (73.6)	0.571
	Moderate Access	98 (9.1)	239 (8.7)	
	Low Access	33 (3.1)	79 (2.9)	
	Care Desert	176 (16.4)	409 (14.8)	
Delivery facility neonatal level	I	97 (9.0)	247 (9.0)	0.671
	II	219 (20.4)	598 (21.7)	
	III	759 (70.6)	1,913 (69.4)	
Birth triage category	Risk-Appropriate	608 (56.6)	1,547 (56.1)	<0.001
	Over-Triage	117 (10.9)	884 (32.1)	
	Under-Triage	350 (32.6)	327 (11.9)	

Values are reported as median [IQR] for continuous variables and *n* (%) for categorical variables. *P* values are descriptive baseline comparisons between early neonatal deaths and surviving matched controls, calculated using Mann–Whitney *U* tests for continuous variables and chi-square tests for categorical variables. Parity was approximated as child birth order minus one.

### 3.2 Temporal trends in early neonatal mortality

Across the study period, early neonatal mortality averaged 3.54 per 1,000 live births and exceeded both late neonatal (1.00 per 1,000) and postneonatal mortality (2.75 per 1,000). Rates remained relatively stable from 2013 through 2019 (range 2.96–3.50 per 1,000) but increased thereafter, reaching 4.59 per 1,000 in 2023 and remaining elevated at 4.32 per 1,000 in 2024 (Table 2).

**Table 2:** Early, late, and postneonatal mortality rates in Arkansas, 2013–2024.

Year	Total births	Early neonatal		Late neonatal		Post neonatal	
		N	Rate	N	Rate	N	Rate
2013	38,419	122	3.18	30	0.78	95	2.47
2014	37,549	114	3.04	30	0.8	99	2.64
2015	37,356	121	3.24	26	0.7	126	3.37
2016	36,582	128	3.5	38	1.04	129	3.53
2017	35,835	121	3.38	45	1.26	115	3.21
2018	35,645	119	3.34	41	1.15	86	2.41
2019	35,176	104	2.96	40	1.14	94	2.67
2020	34,206	129	3.77	37	1.08	90	2.63
2021	34,987	138	3.94	39	1.11	118	3.37
2022	34,451	110	3.19	39	1.13	95	2.76
2023	34,230	157	4.59	31	0.91	82	2.4
2024	34,281	148	4.32	32	0.93	51	1.49
Avg.	**35,726**	**125.92**	**3** **.** **54**	**35.67**	**1** **.** **00**	**98.33**	**2** **.** **75**

*N* = Count of deaths. Rate = Mortality rate per 1,000 live births.

Averages at the bottom are weighted by annual birth volume.

Early neonatal, 0–6 days; late neonatal, 7–27 days; post neonatal, 28–364 days.

The bold values are the Average Values for each column. The whole raw can have a distinctive format to distinguish itself.

Early neonatal mortality increased from 3.24 per 1,000 live births in 2014–2019 to 4.45 per 1,000 in 2023–2024 (risk ratio, 1.37; 95% CI, 1.20–1.57). Decomposition by gestational-age group suggested that changes in the distribution of births across gestational-age strata accounted for approximately 27% of this increase, whereas increases in mortality within gestational-age strata accounted for approximately 73%. Mortality among infants born before 28 weeks increased from 266.4 to 341.3 per 1,000 births (risk ratio, 1.28; 95% CI, 1.11–1.48) and accounted for approximately two-thirds of the absolute statewide increase. Recorded maternal COVID-19 was not associated with increased early neonatal mortality in 2023–2024 (0.98% among births with an early neonatal death vs. 1.84% among surviving births; Fisher *P* = 0.389), and no early neonatal death had COVID-19 recorded as the underlying cause. Home births accounted for only 8 of the 305 early neonatal deaths in 2023–2024. Mortality increases were most pronounced among births with no recorded prenatal visits or only 1–2 visits.

### 3.3 Early neonatal mortality across clinical risk groups

Mortality increased sharply with clinical severity. Early neonatal mortality reached 78.40 per 1,000 live births among infants requiring Level III care and 225.43 per 1,000 among those requiring Level IV care (Figure 1a). Mortality was highest among extremely low birth weight infants (<1,000 g; 276.85 per 1,000; 95% CI, 261.56–292.68) and extremely preterm infants (<28 weeks; 283.30 per 1,000; 95% CI, 267.79–299.34), declining sharply with increasing gestational age and birth weight to less than 1 per 1,000 among term and normal–birth weight infants (Figures 1b,c).

**Figure 1:**
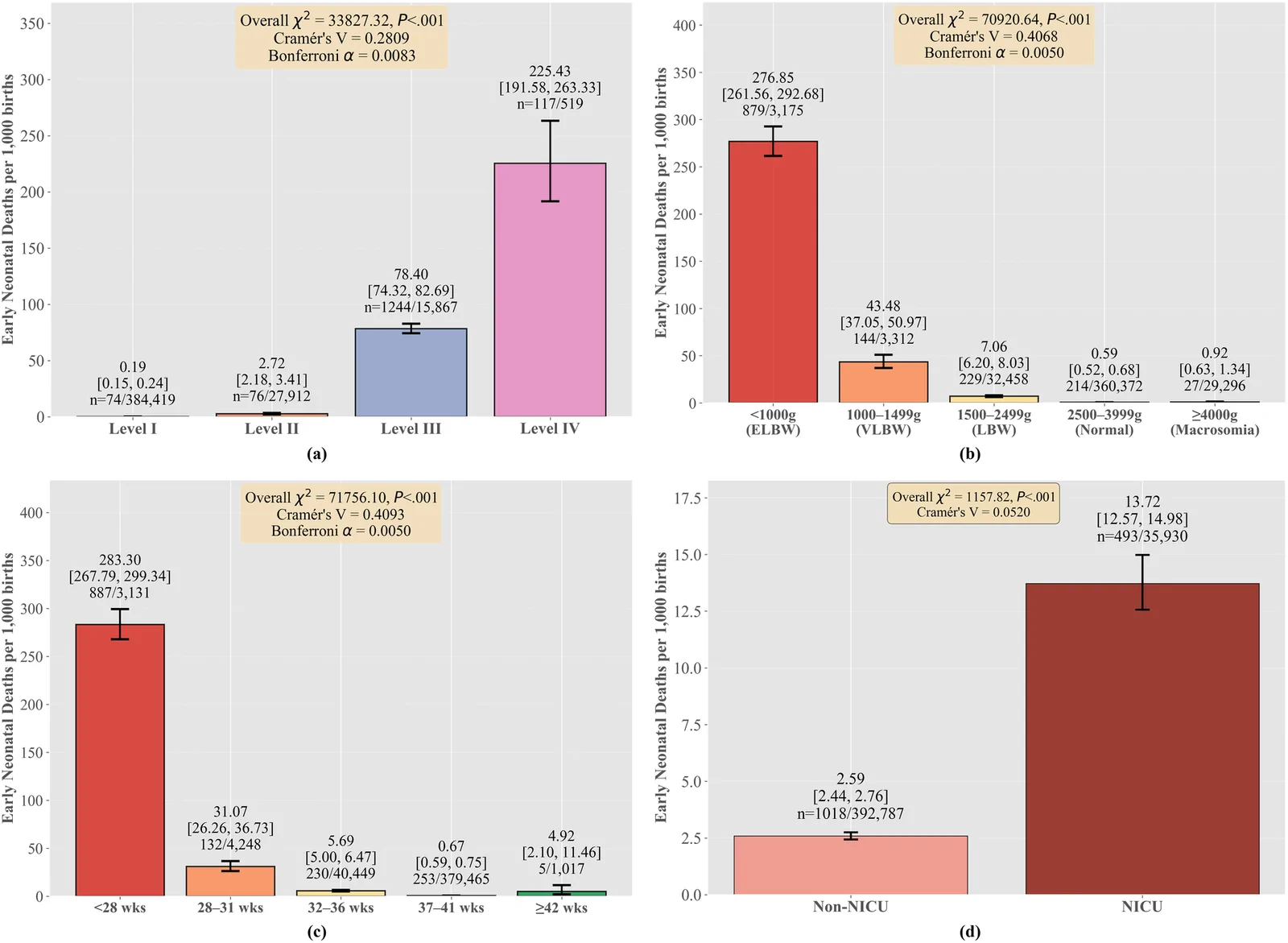
Early neonatal mortality across clinical risk groups. Early neonatal mortality rates per 1,000 births according to **(a)** required neonatal care level, **(b)** birth weight category, **(c)** gestational age category, and **(d)** NICU admission status. *Note. Error bars indicate Wilson 95% confidence intervals. Values above the bars indicate the mortality rate, 95% confidence interval, and number of deaths/total births. Overall associations were evaluated using chi-square tests; Cramér's V indicates effect size, and Bonferroni-adjusted α values were used for pairwise comparisons*.

Among infants admitted to the NICU, overall mortality remained elevated (Figure 1d). Infants requiring interfacility transfer had substantially higher mortality than non-transferred infants (16.51 vs. 3.22 per 1,000; *P* < 0.001), reflecting the concentration of clinically unstable infants within the transferred cohort (Supplementary Figure S1).

### 3.4 Triage alignment and early neonatal mortality

#### 3.4.1 Descriptive associations

Triage alignment was strongly associated with early neonatal mortality across the perinatal care continuum (*P* < 0.001). At birth, under-triage was associated with approximately five-fold higher mortality compared with risk-appropriate care (56.89 vs. 10.70 per 1,000 live births; Figure 2a). Over-triaged infants had the lowest mortality (0.39 per 1,000).

**Figure 2:**
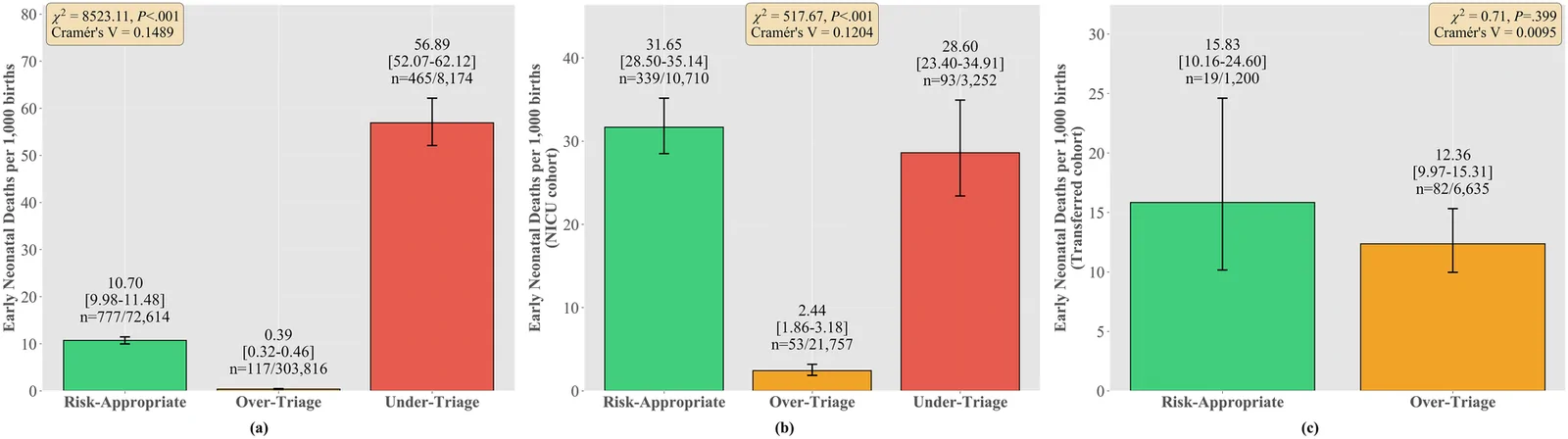
Early neonatal mortality by triage alignment across the perinatal care continuum. Early neonatal mortality rates per 1,000 births according to triage alignment among **(a)** the full study cohort, **(b)** infants admitted to the neonatal intensive care unit, and **(c)** transferred infants. *Note: Under-triaged infants in the transferred cohort were excluded (2 early neonatal deaths among 30 cases) because the estimate would be unstable. Error bars indicate Wilson 95% confidence intervals. Values above the bars indicate the early neonatal mortality rate, 95% confidence interval, and number of deaths/total births. Overall associations were evaluated using chi-square tests, with Cramér's V reported as the effect-size measure.*

Elevated mortality associated with under-triage extended beyond traditionally high-risk neonatal populations. Among term infants, mortality was 17.59 per 1,000 in under-triaged deliveries compared with 1.44 per 1,000 in risk-appropriate settings (*P* < 0.001). Among normal–birth weight infants, mortality was 12.04 vs. 1.30 per 1,000 (*P* < 0.001). These patterns were consistent across all gestational age and birth weight strata (Supplementary Tables S1, S2).

Among NICU-admitted infants, mortality was lowest among over-triaged infants (2.44 per 1,000), while under-triaged and risk-appropriate groups had similarly elevated rates (Figure 2b). Within the transferred cohort, mortality did not differ significantly by transfer alignment (Figure 2c), though mortality remained elevated even among transferred term and normal–birth weight infants, suggesting that transfer identified a clinically unstable subgroup whose risk was not fully captured by gestational age or birth weight alone.

#### 3.4.2 Multivariable analysis

To evaluate the independent association between triage alignment and early neonatal mortality, matched case–control analyses with conditional logistic regression were performed using a sequential modeling strategy (Table 3). Model 1 included system-level exposures; Model 2 additionally adjusted for gestational age and birth weight, the primary pre-delivery confounders; and Model 3 further incorporated post-delivery clinical indicators that may lie on the causal pathway between under-triage and mortality.

**Table 3:** Sequential conditional logistic regression models for early neonatal death.

Variable	Model 1 OR (95% CI)	*p* value	Model 2 OR (95% CI)	*p* value	Model 3 OR (95% CI)	*p* value
Under-triage	5.89 (4.62–7.51)	<0.001	5.85 (4.43–7.72)	<0.001	1.16 (0.76–1.78)	0.484
Distance *>*30 miles	2.45 (1.93–3.11)	<0.001	2.90 (2.21–3.81)	<0.001	1.45 (1.00–2.10)	0.048
0 prenatal visits	2.02 (1.42–2.85)	<0.001	1.51 (1.01–2.24)	0.043	1.71 (1.01–2.88)	0.045
1–3 prenatal visits	2.39 (1.73–3.32)	<0.001	1.78 (1.22–2.59)	0.003	1.82 (1.11–2.99)	0.018
4–7 prenatal visits	1.15 (0.85–1.54)	0.361	1.01 (0.73–1.40)	0.931	1.01 (0.66–1.56)	0.957
8–10 prenatal visits	0.84 (0.64–1.12)	0.240	0.76 (0.55–1.03)	0.080	0.93 (0.62–1.41)	0.745
Medicaid/self-pay vs. private insurance	0.83 (0.70–1.00)	0.044	0.80 (0.65–0.99)	0.040	0.79 (0.61–1.04)	0.095
Gestational age, per week	–	–	0.62 (0.58–0.68)	<0.001	0.60 (0.54–0.67)	*<* 0.001
Birthweight, per 100 g	–	–	0.82 (0.79–0.86)	<0.001	0.88 (0.82–0.93)	*<* 0.001

Model 1 included under-triage, distance greater than 30 miles, prenatal visit categories, and payment source. Model 2 added continuous gestational age and birthweight scaled per 100 g. Model 3 additionally adjusted for clinical and demographic factors, including 5 min Apgar categories, assisted ventilation, prolonged ventilation, congenital anomaly, seizures, multiple birth, infant sex, and maternal race. Reference groups were risk-appropriate or over-triaged delivery for under-triage, 11 or more prenatal visits for prenatal care, and private insurance for payment source.

OR, odds ratio; CI, confidence interval.

In Model 1, under-triage at birth was the strongest predictor of early neonatal death (OR, 5.89; 95% CI, 4.62–7.51). Distance greater than 30 miles to the delivery facility was independently associated with mortality (OR, 2.45; 95% CI, 1.93–3.11), and prenatal care demonstrated a dose-response relationship, with pregnancies receiving fewer than four prenatal visits showing significantly increased odds of death compared with those receiving ≥11 visits. Medicaid/self-pay coverage was associated with modestly lower odds of mortality than private insurance.

After adjustment for gestational age and birth weight (Model 2), the association between under-triage and early neonatal mortality remained essentially unchanged (OR, 5.85; 95% CI, 4.43–7.72), indicating that the excess risk was not explained by baseline differences in gestational age or birth weight. The association with distance strengthened (OR, 2.90; 95% CI, 2.21–3.81), while the prenatal care dose-response persisted, with elevated risk remaining concentrated among pregnancies with fewer than four prenatal visits.

Further adjustment for post-delivery clinical indicators (Model 3) substantially attenuated the association between under-triage and mortality (OR, 1.16; 95% CI, 0.76–1.78). Low 5 min Apgar scores and congenital anomalies emerged as the strongest predictors of mortality, whereas male sex remained independently associated with increased risk. Other post-delivery clinical variables were not independently associated with mortality after adjustment.

Nested sensitivity analysis demonstrated stable estimates for under-triage between Models 1 and 2, followed by marked attenuation after inclusion of post-delivery clinical indicators in Model 3 (Figure 3). Distance showed a similar pattern, whereas the association between limited prenatal care and mortality remained consistent across all models. Congenital anomalies emerged as one of the strongest predictors, although estimates were imprecise because of sparse events. Medicaid/self-pay coverage remained associated with modestly lower adjusted odds of mortality across models (Table 3).

**Figure 3:**
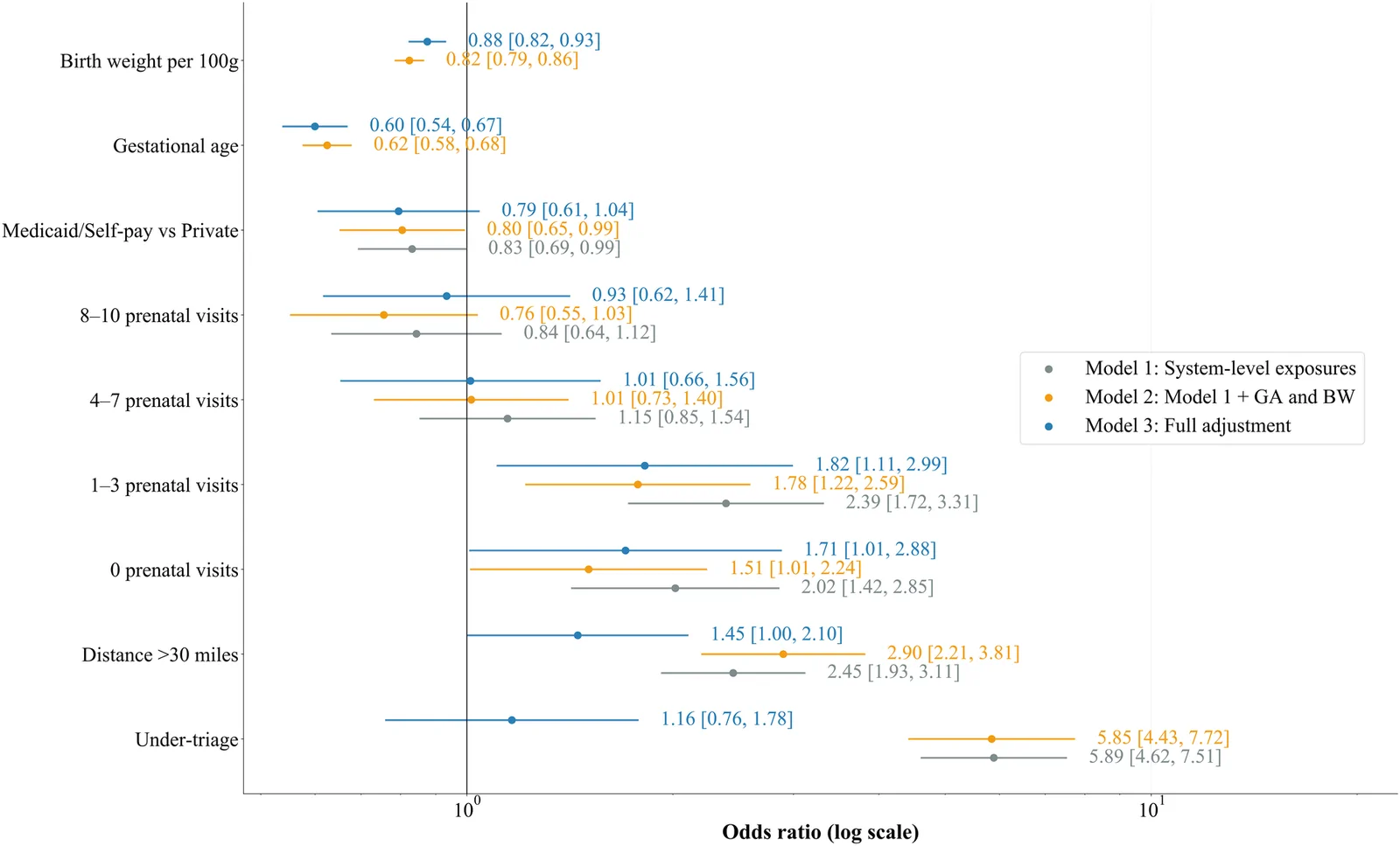
Nested sensitivity analysis of sequential logistic regression models for early neonatal death. Points indicate estimated odds ratios, and horizontal lines indicate 95% confidence intervals. Model 1 included system-level exposures; Model 2 additionally adjusted for gestational age and birth weight; and Model 3 included the full covariate adjustment set. The vertical reference line indicates an odds ratio of 1.0.

As a supplementary prediction-oriented analysis, XGBoost showed good discrimination for early neonatal mortality (mean AUC, 0.901; SD, 0.013; Brier score, 0.104; SD, 0.006). SHAP analysis indicated that prediction was driven primarily by biological and clinical severity markers, led by 5 min Apgar score, birth weight, and gestational age. System-level variables, including travel distance, under-triage, and prenatal care, contributed additional but smaller predictive information (see Supplementary Results and Figure S2).

### 3.5 Prenatal care and sociodemographic factors

Prenatal care utilization was strongly associated with early neonatal survival. Infants born to mothers with no or unrecorded prenatal care experienced substantially higher mortality than those receiving prenatal care (14.08 vs. 2.88 per 1,000; *P* < 0.001; Figure 4a). Mortality decreased progressively with increasing prenatal visit frequency, from 21.37–25.33 per 1,000 among pregnancies with 0–4 visits to 0.93 per 1,000 among pregnancies with 11 or more visits (Figure 4b). This dose-response relationship was confirmed in the multivariable analysis, where 0 and 1–3 prenatal visits remained significantly associated with mortality after adjustment for gestational age and birth weight (Table 3; Figure 3).

**Figure 4:**
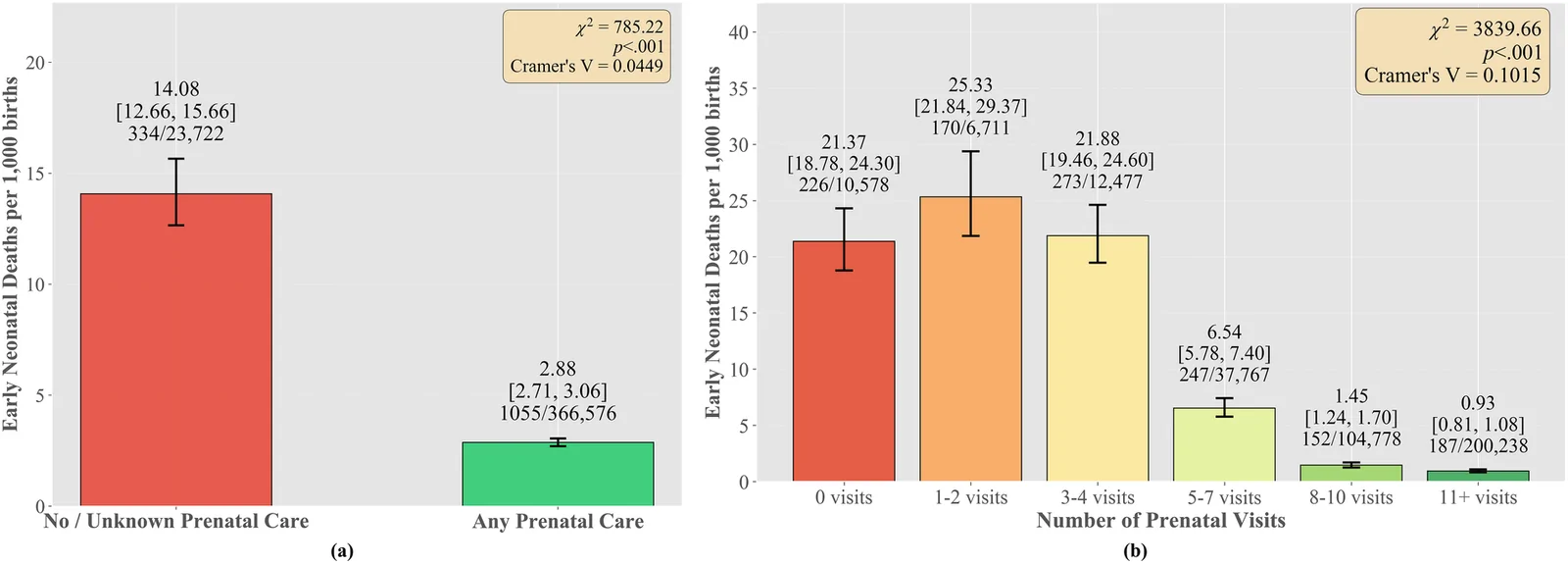
Early neonatal mortality by prenatal care utilization and prenatal visit frequency. Early neonatal mortality rates per 1,000 births according to **(a)** prenatal care status and **(b)** number of prenatal visits. *Note. Panel **(a)** uses the prenatal care status variable and combines births with no recorded prenatal care and births with unknown prenatal care status. Panel **(b)** uses the numeric prenatal visit-count variable; records with missing visit counts were excluded from the visit-frequency categories. Error bars indicate Wilson 95% confidence intervals. Values above the bars indicate the early neonatal mortality rate, 95% confidence interval, and number of deaths/total births. Overall associations were evaluated using chi-square tests, with Cramér's V reported as the effect-size measure.*

Associations between prenatal care and mortality were strongest among term, moderate preterm, normal–birth weight, and large infants, whereas no significant association was observed among extremely preterm or very/extremely low birth weight infants.

Early neonatal mortality differed by maternal race (*P* < 0.001). Infants born to Black/African American mothers had the highest mortality rate (5.49 per 1,000), nearly double that observed among infants born to White mothers (2.90 per 1,000; Supplementary Figure S3). Prenatal care utilization and visit frequency also differed across racial groups, with lower utilization observed among Black/African American and Pacific Islander mothers (Supplementary Figures S4, S5).

Mortality differed by payment source (*P* < 0.001), with modestly higher crude mortality among self-pay births than among privately insured births (Figure 5a). After adjustment, however, Medicaid/self-pay coverage was associated with lower odds of early neonatal death (Model 2: OR 0.80; 95% CI, 0.65–0.99; Table 3). However, this association was attenuated and no longer statistically significant after further adjustment for clinical and demographic characteristics in Model 3 (OR 0.79; 95% CI, 0.61–1.04; *P* = 0.095). Accordingly, payer should not be interpreted as independently protective.

**Figure 5:**
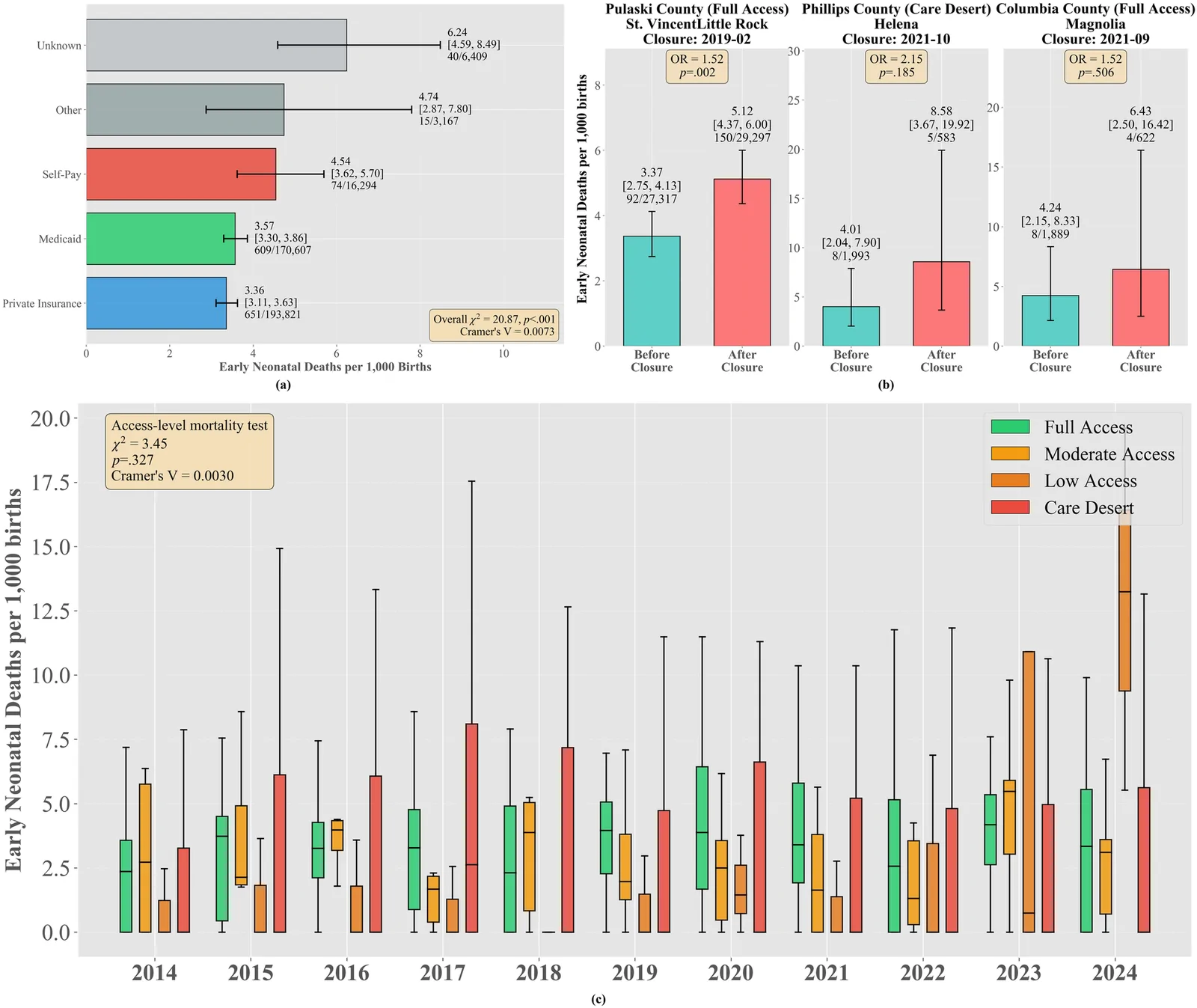
Geographic access, obstetric unit closures, and payment source in relation to early neonatal mortality. Early neonatal mortality rates per 1,000 births according to **(a)** primary payment source, **(b)** the periods before and after selected obstetric unit closures, and **(c)** annual geographic access category from 2014 through 2024. *Note. Error bars in panels **(a)** and **(b)** indicate Wilson 95% confidence intervals. Values shown with the bars indicate the mortality rate, 95% confidence interval, and number of deaths/total births. Panel **(c)** displays annual distributions of county-level early neonatal mortality rates by geographic access category. Overall categorical comparisons are summarized using chi-square tests and Cramér's V; closure comparisons report odds ratios and corresponding p-values.*

### 3.6 Facility closures and geographic access

Between 2019 and 2023, obstetric unit closures were associated with localized increases in early neonatal mortality. In the urban county (County A), mortality increased significantly from 3.37 to 5.12 per 1,000 (OR 1.52; *P* = 0.002). Following rural closures, mortality rates trended upward—more than doubling in one care desert (County B) from 4.01 to 8.58 per 1,000 (OR 2.15; *P* = 0.185)—though these rural shifts lacked statistical significance due to small post-closure event counts (Figure 5b). These localized increases exceeded contemporaneous statewide rates despite relative temporal stability at the broader population level.

At the population level, early neonatal mortality did not differ significantly across county-level maternity care access categories (*χ*^2^ = 3.45; *P* = 0.327; Figure 5c). However, substantial geographic heterogeneity was observed. Seven of the 10 counties with the highest early neonatal mortality rates lacked adequate maternity care access, including 6 maternity care desert counties.

In the multivariable analysis, distance >30 miles to the delivery facility was independently associated with early neonatal death in both Model 1 (OR 2.45; 95% CI, 1.93–3.11) and Model 2 (OR 2.90; 95% CI, 2.21–3.81). The association strengthened from Model 1 to Model 2 after adjustment for gestational age and birth weight (Figure 3). Results were similar when the analysis was restricted to Arkansas residents (Model 2 OR, 2.96; 95% CI, 2.24–3.89; Model 3 OR, 1.54; 95% CI, 1.06–2.25), compared with the corresponding full-cohort estimates (Model 2 OR, 2.90; 95% CI, 2.21–3.81; Model 3 OR, 1.45; 95% CI, 1.00–2.10). These findings support the robustness of the distance association, although Arkansas residents who delivered outside the state were not captured.

### 3.7 Discussion

In this population-based study of a regionalized perinatal system experiencing obstetric unit closures, early neonatal mortality was strongly associated with triage alignment across the perinatal care continuum. After adjustment for gestational age and birth weight, under-triage at birth remained the strongest independent predictor of early neonatal death (Model 2 OR 5.85; 95% CI, 4.43–7.72). Elevated risk extended beyond traditionally high-risk neonatal populations to term and normal-birth-weight infants, with mortality rates more than ten-fold higher in under-triaged vs. risk-appropriate deliveries (e.g., 17.59 vs. 1.44 per 1,000 among term infants).

These findings build on and extend prior evidence on perinatal regionalization. Meta-analysis by Lasswell et al. demonstrated substantially higher mortality for very low birth weight infants born outside Level III centers, with even larger effects among extremely low birth weight infants, and Jensen and Lorch showed that NICU level and volume independently predict morbidity and mortality among very low birth weight infants (6, 7). Walther et al. and Ali et al. further documented pooled benefits of regionalization programs for neonatal and perinatal mortality (37, 38). However, these studies focus primarily on extremely preterm and very low birth-weight populations and evaluate delivery hospital or NICU outcomes in isolation. By quantifying the mortality penalty associated with under-triage across the full risk spectrum—including term and normal-birth-weight infants—and across multiple stages of the continuum, our results show that the consequences of misalignment are not confined to the highest-risk groups.

The pattern of mortality across the continuum supports a mechanism in which the most critical harm occurs at the point of delivery. Among NICU-admitted infants, mortality was high across triage categories but converged between under-triaged and risk-appropriate admissions (31.65 vs. 28.60 per 1,000). In the transferred cohort, mortality was substantially higher than in non-transferred infants (16.51 vs. 3.22 per 1,000), yet did not differ significantly by transfer alignment, suggesting that transfer identifies a clinically unstable subgroup whose risk is not fully captured by gestational age or birth weight. These patterns are consistent with prior findings that birth location has a stronger influence on outcomes than postnatal transfer. Helenius et al. showed that extremely preterm infants born in non-tertiary hospitals and transferred within 48 h had higher odds of severe brain injury compared with those born in tertiary settings, and Mori et al. reported that transport durations exceeding 90 min were associated with more than twice the rate of neonatal death (8, 9). Together, this evidence suggests that under-triage exerts its effect primarily through delays in initial stabilization and resuscitation, rather than through ongoing differences in NICU-level care.

### 3.8 Sequential modeling, mediation, and overadjustment

A central methodological contribution of this study is the explicit distinction between pre-delivery confounders and post-delivery variables that may lie on the causal pathway between under-triage and death. The sequential modeling strategy and nested sensitivity analysis were designed to address this issue, which is particularly salient in perinatal epidemiology where many clinically important variables are both markers of severity and potential consequences of earlier care.

Model 2, which adjusts for gestational age and birth weight alongside system-level exposures, represents the primary causal estimate. Gestational age and birth weight are determined before the triage exposure (delivery location) and independently predict both facility choice and neonatal mortality; they are therefore legitimate confounders requiring adjustment (6, 7). The nested sensitivity analysis shows that the under-triage odds ratio was essentially unchanged from Model 1 (5.89) to Model 2 (5.85), confirming that the under-triage effect is not simply a proxy for clinical severity and it persists robustly after accounting for the two strongest clinical risk factors for neonatal death.

In contrast, when post-delivery clinical indicators (5 min Apgar score, assisted and prolonged ventilation, seizures, congenital anomaly, and infant sex) were added in Model 3, the under-triage association was attenuated to non-significance (OR 1.16; 95% CI, 0.76–1.78). The nested sensitivity analysis visualizes this attenuation pattern, demonstrating that the shift occurs specifically when post-delivery variables are introduced. This behavior is consistent with overadjustment for mediators rather than correction of residual confounding. Schisterman et al. and Richiardi et al. have highlighted how adjusting for variables on the causal pathway can attenuate or even reverse true exposure effects in observational studies, and Ananth and Brandt discussed similar concerns in the context of perinatal mediation analysis (32–34). Directed acyclic graphs tailored to perinatal outcomes similarly emphasize the importance of distinguishing confounding from mediation when deciding which variables to adjust for (35, 36).

Clinically, many of the Model 3 variables are plausible mediators. Low Apgar scores, the need for ventilation, and seizures can reflect delayed or inadequate resuscitation and stabilization, processes that may be compromised when high-risk infants are delivered in under-resourced facilities (30, 31). Adjusting for these variables therefore “blocks” part of the causal pathway from under-triage to death, which explains why their inclusion attenuates the under-triage effect. For interpretation of triage misalignment as a system-level determinant of mortality, Model 2—which accounts for intrinsic risk but preserves this pathway—provides the more appropriate estimate.

The supplementary XGBoost analysis provided a complementary prediction-oriented view of the same pattern, showing that biological severity dominated mortality prediction while prenatal care, travel distance, payer, and under-triage added smaller incremental information. Because SHAP values reflect predictive contribution rather than causal effects, these findings should be interpreted as supportive of, rather than independent from, the matched conditional logistic regression results.

### 3.9 Prenatal care as a system-level determinant

Prenatal care utilization showed a strong dose–response relationship with early neonatal survival, both descriptively and in multivariable models. Infants born to mothers with no or unrecorded prenatal care experienced substantially higher mortality than those receiving care (14.08 vs. 2.88 per 1,000), and mortality decreased progressively with increasing prenatal visit frequency: from more than 20 per 1,000 among pregnancies with 0–4 visits to less than 1 per 1,000 among pregnancies with ≥11 visits. In the conditional logistic regression, categories with 0 and 1–3 prenatal visits remained significantly associated with mortality after adjustment for gestational age and birth weight, whereas intermediate visit bands did not differ from the high-visit reference. Notably, the sharpest reduction in mortality occurred between the lowest visit bands and approximately 5–7 visits, after which additional visits yielded diminishing marginal reductions. This inflection point is broadly consistent with the 2025 ACOG Clinical Consensus on tailored prenatal care, which supports targeted schedules of roughly 6–10 visits for average-risk individuals based on systematic reviews showing equivalent maternal and neonatal outcomes with reduced visit frequency in high-income settings, and with WHO guidance that very sparse schedules (around four goal-oriented visits) may be associated with borderline increases in perinatal mortality in some low- and middle-income contexts (39, 40). Within this framework, our findings suggest that the critical system-level function of prenatal care in a regionalized perinatal network is not simply accumulating visits, but achieving a minimum threshold of engagement that enables early risk identification, referral, and planned delivery at appropriately resourced facilities. Pregnancies with fewer than four visits, which fall well below even the most reduced evidence-based schedules, appear to represent a qualitatively different pattern of care engagement in which the system's capacity for risk identification and triage alignment is fundamentally compromised.

These findings align with prior work showing that greater antenatal care access and intensity are associated with lower neonatal mortality (10, 11, 41, 42). Within a regionalized perinatal system, however, prenatal care plays an additional role as an upstream determinant of triage alignment rather than only an individual-level risk factor. Adequate prenatal care functions as the system's “triage engine” by supporting early risk identification, referral, and planned delivery in appropriately resourced facilities. Conversely, limited prenatal care may delay recognition of risk until the intrapartum or postnatal period, when *in utero* transfer is no longer possible and stabilization must occur after delivery at lower-level facilities, where under-triage carries the greatest mortality penalty.

The stronger associations observed among term, moderate preterm, normal-birth-weight, and large infants, but not among extremely preterm or extremely low-birth-weight infants, are consistent with this mechanism. Among the highest-risk infants, intrinsic vulnerability may dominate mortality risk, whereas among lower-risk infants, missed opportunities for timely identification and referral may be more consequential. In this sense, prenatal care serves as a system-level gatekeeper for triage alignment.

The observed racial differences in early neonatal mortality and prenatal care utilization further highlight the importance of upstream system factors. Infants born to Black/African American mothers had nearly double the early neonatal mortality rate of those born to White mothers, and prenatal care utilization and visit frequency were lower among Black/African American and Pacific Islander mothers. These patterns echo broader literature documenting racial and ethnic disparities in perinatal outcomes and access to high-quality care and suggest that inequities in prenatal care and referral pathways may translate directly into inequities in triage alignment and early neonatal survival within regionalized systems (43, 44).

Exploratory analyses of the 2023–2024 increase did not identify a single explanatory factor. Recorded maternal or neonatal COVID-19 did not account for the increase, and although home births became more frequent, they represented only a small fraction of early neonatal deaths. In contrast, mortality was higher among infants born at <28 weeks and among pregnancies with very limited prenatal care. These findings suggest that the recent increase may reflect a combination of clinical severity and vulnerabilities in access and care pathways rather than a single cause.

### 3.10 Geographic barriers, obstetric unit closures, and localized mortality

Geographic access and obstetric unit closures emerged as important system-level determinants of early neonatal mortality. At the population level, early neonatal mortality did not differ significantly across county-level maternity care access categories, suggesting relative temporal stability in aggregate statewide rates. However, substantial geographic heterogeneity was evident: seven of the ten counties with the highest early neonatal mortality rates lacked adequate maternity care access, including six classified as maternity care deserts.

Within counties experiencing obstetric unit closures, we observed localized increases in early neonatal mortality that were not apparent at the statewide level. In one urban county, mortality increased from 3.37 to 5.12 per 1,000 after closure (OR 1.52; *P* = 0.002), and in a rural care-desert county mortality more than doubled from 4.01 to 8.58 per 1,000, although the latter change did not reach statistical significance due to small numbers (OR 2.15; *P* = 0.185). These localized shifts exceeded contemporaneous statewide rates, suggesting that localized changes temporally associated with closures can be masked when outcomes are monitored only at aggregate levels. Prior studies have shown that closures can have heterogeneous effects: Kozhimannil et al. found that loss of rural obstetric services was associated with increases in out-of-hospital births, births in hospitals without obstetric units, and preterm births, whereas Wesselius et al. reported decreased perinatal mortality after closure of an urban community hospital obstetric unit, attributed to centralization and volume effects (17, 20). Malouf et al.'s systematic review and Durrance et al.'s analysis of rural hospital closures similarly emphasize that the impact of closures depends on alternative facility availability, travel distances, and referral network integrity (21, 22).

Distance to the delivery facility was independently associated with early neonatal death, and this association strengthened after adjustment for gestational age and birth weight. In Model 1, distance >30 miles was associated with increased odds of death (OR 2.45; 95% CI, 1.93–3.11); in Model 2, the association increased further (OR 2.90; 95% CI, 2.21–3.81). One plausible explanation is that unadjusted estimates partially reflect selective referral of higher-acuity pregnancies to distant tertiary centers, whereas adjustment for clinical severity reveals a larger independent association between geographic access and mortality. This interpretation is consistent with prior studies linking longer travel times and greater distance to delivery facilities with adverse perinatal and child health outcomes (45, 46).

The paradoxical protective association of Medicaid/self-pay coverage in multivariable models should be interpreted cautiously. Although crude mortality was lowest among privately insured births, Medicaid/self-pay was associated with modestly lower odds of mortality in Model 2, but this association was attenuated and no longer statistically significant after additional adjustment in Model 3. This pattern suggests that the apparent association reflects differences in clinical, demographic, and access-related characteristics across payer groups rather than an independently protective effect of Medicaid or self-pay coverage.

### 3.11 Implications for regionalized perinatal systems

Taken together, these findings have several concrete implications for the design and monitoring of regionalized perinatal systems. First, triage misalignment at birth should be treated as a core performance indicator, not a niche concern confined to extremely preterm or very low-birth-weight infants. Our data show that under-triage is a major determinant of early neonatal death across the risk spectrum, including among term and normal-birth-weight infants who are not usually targeted in regionalization metrics. Ensuring that infants whose clinical trajectories may rapidly deteriorate, whether due to unanticipated intrapartum complications or congenital anomalies, are delivered in facilities capable of timely stabilization and escalation is essential for reducing preventable mortality. Performance monitoring in regionalized systems should therefore incorporate explicit measures of triage alignment at birth (e.g., proportion of high-risk and “unexpected high-risk” infants delivered below required level of care), and these metrics should be applied beyond the VLBW/extremely preterm population.

Second, prenatal care should be recognized and measured as the upstream determinant of triage alignment. Prenatal care emerges in this study as both a traditional determinant of neonatal health and the primary mechanism by which systems identify high-risk pregnancies, plan deliveries at higher-level centers, and operationalize *in utero* transfer. The dose-response relationship, with the sharpest reduction in mortality occurring between low visit bands and achieving ≥5 visits, suggests that ensuring adequate prenatal care engagement, particularly reaching a minimum threshold of visits, is a modifiable system-level intervention to improve triage alignment. Interventions to increase prenatal care coverage and visit intensity in high-risk and underserved populations, and to standardize risk assessment and referral protocols within prenatal care, should be priorities for regionalization efforts (41, 42). In practice, this means that perinatal quality collaboratives and payers should treat missed or low-intensity prenatal care not only as a clinical risk but as a signal of potential triage failure, with targeted outreach and referral pathways designed to close that gap.

Third, quality measurement and risk-adjusted benchmarking in regionalized systems must distinguish pre-delivery confounders from post-delivery mediators. Conventional “fully adjusted” models that include Apgar scores, ventilation, seizures, and other post-delivery variables may substantially underestimate the true impact of triage misalignment by adjusting away consequences of care (32–34). For system-level performance assessment and policy decisions, such as evaluating regionalization initiatives, obstetric unit closures, or NICU verification, models that adjust for intrinsic risk (gestational age, birth weight, major anomalies) but preserve pathways through stabilization and resuscitation are more appropriate. Incorporating this perspective into quality metrics, as advocated by Iriye et al. and in ACOG/SMFM Levels of Maternal Care consensus statements, would better align analytic practices with the goals of risk-appropriate care (47, 48).

Fourth, evaluations of obstetric unit closures and service consolidation should explicitly incorporate neonatal outcomes and triage pathways at a granular level. The localized mortality increases we observed after closures, despite stable statewide early neonatal mortality, indicate that closure-related vulnerabilities can be obscured by aggregate indicators. State-level risk-appropriate care policies, perinatal quality collaboratives, and regionalization plans should include mechanisms to detect and respond to county-level or facility-level increases in early neonatal deaths, especially in maternity care deserts and rural counties with limited alternative facilities, rather than relying solely on statewide rates (17, 21, 22). In this sense, early neonatal death functions as a stress test for regionalized perinatal systems, revealing vulnerabilities in triage, access, prenatal risk identification, and coordination that might otherwise remain hidden.

Finally, integrating these elements-triage alignment metrics, prenatal care thresholds and referral protocols, distance and closure monitoring, and appropriate risk adjustment-into a single system-level dashboard would provide a practical framework for ongoing performance review. Such a framework could be implemented through existing infrastructures like state perinatal quality collaboratives, supporting routine identification of triage misalignment events and targeted interventions to strengthen risk-appropriate care for mothers and infants.

### 3.12 Limitations

This study has several limitations that should be considered when interpreting the findings. First, classification of required level of neonatal care and triage status relied on variables available in linked vital records and administrative data rather than detailed clinical charts. Gestational age, birth weight, Apgar scores, ventilation indicators, and anomaly flags may not fully capture clinical complexity, and misclassification of required level or facility level could occur. Any non-differential misclassification of triage alignment would be expected to attenuate associations toward the null, suggesting that the true impact of under-triage may be larger than observed. Second, geographic access measures were based on county-level classifications and straight-line distance from the maternal county centroid to the delivery facility, treated as static over time. These measures may not reflect within-county heterogeneity, temporal changes in local access, or patient-specific travel routes and times. As a result, our estimates of distance and maternity care desert status may underestimate fine-grained access barriers. Third, analyses involving obstetric unit closures were limited by small sample sizes within affected counties. Post-closure event counts were particularly sparse in rural maternity care deserts, reducing statistical power to detect differences and yielding wide confidence intervals. These analyses should therefore be interpreted as hypothesis-generating rather than definitive estimates of closure effects. Fourth, transfer analyses were restricted to interfacility transfers involving hospitals with known neonatal care levels. Under-triaged transfers were too uncommon for stable inferential analysis and were excluded, which conservatively biases the transfer-triage association toward the null. In addition, infants who die before transfer can be initiated are not captured in the transferred cohort, introducing potential survival bias that may underestimate the true mortality burden associated with birth at under-resourced facilities. Fifth, although the matched case-control design is appropriate for rare outcomes such as early neonatal death, residual confounding from unmeasured variables remains possible. We adjusted for major clinical and system-level factors, but unmeasured differences in referral decision-making, intrapartum events, and facility-specific practices may still have influenced the observed associations. Sixth, the interpretation that post-delivery clinical indicators mediate part of the association between under-triage and mortality is based on causal reasoning supported by sequential modeling and nested sensitivity analyses rather than formal causal mediation methods. We did not perform a pre-specified mediation analysis to quantify the proportion of the under-triage effect operating through clinical deterioration pathways. Future studies applying formal causal mediation methods could better quantify the contribution of Apgar depression, ventilation requirements, and other post-delivery indicators. Finally, this study was conducted within a single state's regionalized perinatal system during a period of changing obstetric capacity. System organization, policy context, and baseline access patterns may differ in other settings; therefore, generalizability to states with different regionalization structures, urban-rural distributions, or risk-appropriate care policies may be limited. As with all observational studies, causal relationships cannot be definitively established, and these findings should be interpreted alongside evidence from other regionalized perinatal systems.

## 4 Conclusions

In this population-based study of a regionalized perinatal system, under-triage at birth emerged as the strongest independent predictor of early neonatal death after adjustment for gestational age and birth weight, with excess risk extending beyond traditionally high-risk populations to include term and normal-birth-weight infants. Prenatal care intensity, geographic access, and structural changes in obstetric capacity influenced mortality through their effects on triage alignment and timely access to risk-appropriate care, while attenuation of the under-triage association after adjustment for post-delivery clinical indicators supports the hypothesis that delayed stabilization and escalation mediate part of this excess risk.

Taken together, these findings suggest that regionalized perinatal care should be viewed not simply as a hierarchy of hospitals, but as an integrated system whose effectiveness depends on successful movement through sequential processes of prenatal risk identification, triage alignment, risk-appropriate delivery, timely stabilization, escalation of care, and coordinated referral and transfer. Within this framework, under-triage represents not merely a mismatch between patient and facility, but a measurable indicator of system coordination across the continuum of care.

These findings have important implications for regionalized perinatal systems. Performance monitoring should extend beyond traditional metrics focused on very preterm and very low-birth-weight infants to include triage alignment across the broader neonatal population. Prenatal care engagement, geographic access, and localized changes in obstetric capacity should be recognized as system-level determinants of risk-appropriate care and incorporated into quality improvement efforts. Monitoring triage alignment at birth, prenatal care intensity, distance-related access barriers, and localized mortality following structural changes may help identify preventable early neonatal deaths and guide policies that strengthen regionalized systems of care for mothers and infants.

## Data Availability

The raw data supporting the conclusions of this article will be made available by the authors, without undue reservation.

## References

[1] Lehtonen L Gimeno A Parra-Llorca A Vento M. Early neonatal death: a challenge worldwide. Semin Fetal Neonatal Med. (2017) 22(3):153–60. 10.1016/j.siny.2017.02.006 28238633

[2] Lawn JE Blencowe H Oza S You D Lee ACC Waiswa P. Every newborn: progress, priorities, and potential beyond survival. Lancet. (2014) 384(9938):189–205. 10.1016/S0140-6736(14)60496-7 24853593

[3] Oza S Lawn JE Hogan DR Mathers C Cousens SN. Neonatal cause-of-death estimates for the early and late neonatal periods for 194 countries: 2000–2013. Bull World Health Organ. (2015) 93(1):19–28. 10.2471/BLT.14.139790 25558104 PMC4271684

[4] March of Dimes. Toward Improving the Outcome of Pregnancy: Recommendations for the Regional Development of Maternal and Perinatal Health Services. White Plains (NY): March of Dimes National Foundation (1976).

[5] Stark AR Pursley DWM Papile L-A Eichenwald EC Hankins CT Buck RK. Standards for levels of neonatal care: II, III, and IV. Pediatrics. (2023) 151(6):e2023061957. 10.1542/peds.2023-061957 37212022

[6] Lasswell SM Barfield WD Rochat RW Blackmon L. Perinatal regionalization for very low-birth-weight and very preterm infants: a meta-analysis. JAMA. (2010) 304(9):992–1000. 10.1001/jama.2010.1226 20810377

[7] Jensen EA Lorch SA. Effects of a birth hospital’s neonatal intensive care unit level and annual volume of very low-birth-weight infant deliveries on morbidity and mortality. JAMA Pediatr. (2015) 169(8):e151906. 10.1001/jamapediatrics.2015.1906 26237466 PMC11528919

[8] Helenius K Longford N Lehtonen L Modi N Gale C. Association of early postnatal transfer and birth outside a tertiary hospital with mortality and severe brain injury in extremely preterm infants: observational cohort study with propensity score matching. Br Med J. (2019) 367:l5678. 10.1136/bmj.l5678 PMC6812621 31619384

[9] Mori R Fujimura M Shiraishi J Evans B Corkett M Negishi H. Duration of inter-facility neonatal transport and neonatal mortality: systematic review and cohort study. Pediatr Int. (2007) 49(4):452–8. 10.1111/j.1442-200X.2007.02393.x 17587267

[10] McElfish PA Caldwell AR Selig JP Gomez Pomar E Hawley NL Willis DE. Examining the benefits of early prenatal care using US birth records. Public Health Rep. (2026) 13. 10.1177/00333549261436733 PMC13171713 42124551

[11] Partridge S Balayla J Holcroft CA Abenhaim HA. Inadequate prenatal care utilization and risks of infant mortality and poor birth outcome: a retrospective analysis of 28,729,765 U.S. Deliveries over 8 years. Am J Perinatol. (2012) 29(10):787–93. 10.1055/s-0032-1316439 22836820

[12] Harrison W Goodman D. Epidemiologic trends in neonatal intensive care, 2007–2012. JAMA Pediatr. (2015) 169(9):855–62. 10.1001/jamapediatrics.2015.1305 26214387

[13] Boghossian NS Geraci M Phibbs CS Lorch SA Edwards EM Horbar JD. Trends in resources for neonatal intensive care at delivery hospitals for infants born younger than 30 weeks’ gestation, 2009–2020. JAMA Netw Open. (2023) 6(5):e2312107. 10.1001/jamanetworkopen.2023.12107 37145593 PMC10163386

[14] Kroelinger CD Okoroh EM Goodman DA Lasswell SM Barfield WD. Comparison of state risk-appropriate neonatal care policies with the 2012 AAP policy statement. J Perinatol. (2018) 38(4):411–20. 10.1038/s41372-017-0006-6 29209032 PMC5955781

[15] Kroelinger CD Rice ME Okoroh EM DeSisto CL Barfield WD. Seven years later: state neonatal risk-appropriate care policy consistency with the 2012 American academy of pediatrics policy. J Perinatol. (2022) 42(5):595–602. 10.1038/s41372-021-01146-y 34253843 PMC9198846

[16] Wilkers JL DeSisto CL Ewing AC. Levels of neonatal care among birth facilities in 20 states and other jurisdictions: CDC levels of care assessment tool (CDC LOCATe). J Perinatol. (2023) 43(10):1283–90. 10.1038/s41372-023-01741-9 PMC10230433 36138088

[17] Kozhimannil KB Hung P Henning-Smith C Casey MM Prasad S. Association between loss of hospital-based obstetric services and birth outcomes in rural counties in the United States. JAMA. (2018) 319(12):1239–47. 10.1001/jama.2018.1830 29522161 PMC5885848

[18] Kozhimannil KB Sheffield EC Busse CE Interrante JD McDaniel CE Handley SC. Availability of higher-level neonatal care in rural and urban US hospitals, 2010–2022. JAMA Netw Open. (2026) 9(2):e2559680. 10.1001/jamanetworkopen.2025.59680 41678195 PMC12902888

[19] Nasrollahi SM Meale SJ Morgavi DP Schiphorst AM Robins RJ Ortigues-Marty I. Out-of-maternity deliveries in France: a nationwide population-based study. PLoS One. (2020) 15(6):e0234344. 10.1371/journal.pone.0234344 32502191 PMC7274422

[20] Haavisto H Rinne K Kolari T Anttila E Ojala E Polo-Kantola P. Decrease in perinatal mortality after closure of obstetric services in a community hospital in Amsterdam, The Netherlands: a retrospective cohort study. Eur J Obstet Gynecol Reprod Biol. (2023) 283:25–31. 10.1016/j.ejogrb.2023.01.028 36753902

[21] Malouf RS Tomlinson C Henderson J Opondo C Brocklehurst P Alderdice F. Impact of obstetric unit closures, travel time and distance to obstetric services on maternal and neonatal outcomes in high-income countries: a systematic review. BMJ Open. (2020) 10(12):e036852. 10.1136/bmjopen-2020-036852 33318106 PMC7735086

[22] Ellis W Rawlinson K Milstein B Erickson J Creegan A Lanford D. The effect of rural hospital closures on maternal and infant health. Health Serv Res. (2024) 59(2):e14267. 10.1111/1475-6773.14267 37840011 PMC10915477

[23] Sharon MAK Eksioglu SD Bogulski CA Gomez Pomar E. Inequitable access to risk-appropriate neonatal care: evidence from a regionalized perinatal system in Arkansas. Front Reprod Health. (2026) 8:1914178. 10.3389/frph.2026.1914178 42666497 PMC13522216

[24] National Center for Health Statistics. List of all valid ICD-10 codes, 1999-2022: including both 3-digit and 4-digit codes and titles. Centers for Disease Control and Prevention. Available online at: https://ftp.cdc.gov/pub/health_statistics/nchs/publications/ICD10/allvalid2020%20(detailed_titles_headings).pdf (Accessed April 11, 2026).

[25] Arkansas Department of Health. Perinatal regionalization—Arkansas Maternal and Perinatal Outcomes Quality Review Committee. Available online at: https://healthy.arkansas.gov/boards-commissions/committees/arkansas-maternal-perinatal-outcomes-quality-review-committee/perinatal-regionalization/ (Accessed November 29, 2025).

[26] Arkansas Center for Health Improvement. Arkansas birthing hospitals. Available online at: https://achi.net/maternal-infant-health/#arkansas-birthing-hospitals (Accessed November 29, 2025).

[27] U.S. Maternal Health Portal. Arkansas: maternity care access. Available online at: https://ar.maternalhealth.us/maternity-care-access (Accessed November 29, 2025).

[28] Sun JX Sinha S Wang S Maiti T. Bias reduction in conditional logistic regression. Stat Med. (2011) 30(4):348–55. 10.1002/sim.4105 21225897

[29] Puhr R Heinze G Nold M Lusa L Geroldinger A. Firth’s logistic regression with rare events: accurate effect estimates and predictions? Stat Med. (2017) 36(14):2302–17. 10.1002/sim.7273 28295456

[30] Cnattingius S Johansson S Razaz N. Apgar score and risk of neonatal death among preterm infants. N Engl J Med. (2020) 383(1):49–57. 10.1056/NEJMoa1915075 32609981

[31] Committee on Obstetric Practice. Committee opinion no. 644: the apgar score. Obstet Gynecol. (2015) 126(4):e52–5. 10.1097/AOG.0000000000001108 26393460

[32] Schisterman EF Cole SR Platt RW. Overadjustment bias and unnecessary adjustment in epidemiologic studies. Epidemiology. (2009) 20(4):488–95. 10.1097/EDE.0b013e3181a819a1 19525685 PMC2744485

[33] Richiardi L Bellocco R Zugna D. Mediation analysis in epidemiology: methods, interpretation and bias. Int J Epidemiol. (2013) 42(5):1511–9. 10.1093/ije/dyt127 24019424

[34] Ananth CV Brandt JS. A principled approach to mediation analysis in perinatal epidemiology. Am J Obstet Gynecol. (2022) 226(1):24–32.e6. 10.1016/j.ajog.2021.10.028 34991898 PMC9204564

[35] Bandoli G Palmsten K Flores KF Chambers CD. Constructing causal diagrams for common perinatal outcomes: benefits, limitations and motivating examples with maternal antidepressant use in pregnancy. Paediatr Perinat Epidemiol. (2016) 30(5):521–8. 10.1111/ppe.12302 27160789 PMC4970924

[36] Digitale JC Martin JN Glymour MM. Tutorial on directed acyclic graphs. J Clin Epidemiol. (2022) 142:264–7. 10.1016/j.jclinepi.2021.08.001 34371103 PMC8821727

[37] Walther F Küster DB Bieber A Rüdiger M Malzahn J Schmitt J. Impact of regionalisation and case-volume on neonatal and perinatal mortality: an umbrella review. BMJ Open. (2020) 10(9):e037135. 10.1136/bmjopen-2020-037135 32978190 PMC7520832

[38] Ali AA Naseem HA Allahuddin Z Yasin R Azhar M Hanif S. The effectiveness of regionalization of perinatal care and specific facility-based interventions: a systematic review. Neonatology. (2025) 122(Suppl 1):245–61. 10.1159/000541384 39504943 PMC11875419

[39] American College of Obstetricians and Gynecologists Committee on Clinical Consensus—Obstetrics. Tailored Prenatal Care Delivery for Pregnant Individuals: ACOG Clinical Consensus No. 8. Obstet Gynecol. (2025) 145(5):565–77. 10.1097/AOG.0000000000005889 40245426

[40] World Health Organization. WHO Recommendations on Antenatal Care for a Positive Pregnancy Experience. World Health Organization (2016). Available online at: https://iris.who.int/handle/10665/250796 (Accessed July 13, 2026). 28079998

[41] Tekelab T Chojenta C Smith R Loxton D. The impact of antenatal care on neonatal mortality in sub-saharan Africa: a systematic review and meta-analysis. PLoS One. (2019) 14(9):e0222566. 10.1371/journal.pone.0222566 31518365 PMC6743758

[42] Doku DT Neupane S. Survival analysis of the association between antenatal care attendance and neonatal mortality in 57 low- and middle-income countries. Int J Epidemiol. (2017) 46(5):1668–77. 10.1093/ije/dyx125 29040531 PMC5837573

[43] Sheikh J Allotey J Kew T Fernández-Félix BM Zamora J Khalil A. Effects of race and ethnicity on perinatal outcomes in high-income and upper-middle-income countries: an individual participant data meta-analysis of 2,198,655 pregnancies. Lancet. (2022) 400(10368):2049–62. 10.1016/S0140-6736(22)01191-6 36502843

[44] Greenberg MB Gandhi M Davidson C Carter EB. Society for maternal-fetal medicine consult series #62: best practices in equitable care delivery—addressing systemic racism and other social determinants of health as causes of obstetrical disparities. Am J Obstet Gynecol. (2022) 227(2):B44–59. 10.1016/j.ajog.2022.04.001 35378098

[45] Paranjothy S Watkins WJ Rolfe K Adappa R Gong Y Dunstan F. Perinatal outcomes and travel time from home to hospital: Welsh data from 1995 to 2009. Acta Paediatr. (2014) 103(12):e522–7. 10.1111/apa.12800 25197024

[46] Karra M Fink G Canning D. Facility distance and child mortality: a multi-country study of health facility access, service utilization, and child health outcomes. Int J Epidemiol. (2017) 46(3):817–826. 10.1093/ije/dyw062 27185809

[47] Iriye BK Gregory KD Saade GR Grobman WA Brown HL. Quality measures in high-risk pregnancies: executive summary of a cooperative workshop of the society for maternal-fetal medicine, national institute of child health and human development, and the American college of obstetricians and gynecologists. Am J Obstet Gynecol. (2017) 217(4):621–25. 10.1016/j.ajog.2017.07.014 28735702

[48] American College of Obstetricians and Gynecologists and the Society for Maternal-Fetal Medicine. Levels of maternal care: obstetric care consensus no. 9. Obstet Gynecol. (2019) 134(2):e41–55. 10.1097/AOG.0000000000003383 31348224

