# Supplementary material for: Triage misalignment and early neonatal death in regionalized perinatal care
Source: Front Reprod Health. 2026 Oct 13;8:1944917. doi: 10.3389/frph.2026.1944917 (PMC13710249; doi:10.3389/frph.2026.1944917)
Supplement: Supplementary file 1 [file Supplementaryfile1.docx]

# Section 1: Supplementary Methods and Model-Based Analyses

# Record linkage and deduplication

Birth and death records were deterministically linked using a two-step strategy. First, records with valid birth certificate numbers were linked directly by normalized birth certificate number. Second, among birth records not matched in the primary linkage step, remaining records were linked using a composite key based on the child’s unique APCD identifier, infant sex, and maternal county of residence. Linked records from both strategies were combined, and age at death was recalculated using the birth date from the birth record and the death date from the death certificate. Records with negative age at death were excluded. When duplicate links occurred, primary birth-certificate matches were prioritized over secondary APCD-based matches, and records were sorted by linkage priority and death date. The linked death file was then deduplicated by birth certificate number, retaining the first eligible death record for each birth certificate. Death indicators for early neonatal death, late neonatal death, neonatal death, post neonatal death, and infant death were then merged back into the full birth cohort using the normalized birth certificate number.

# Study cohorts and analytic windows

The full 2013–2024 birth cohort was used for statewide mortality time-series analyses, county-level hotspot mapping, clinical risk strata analyses (gestational age, birth weight, and required care level), and obstetric unit closure analyses. Analyses requiring facility-level or administrative variables incompletely captured in 2013 (including triage alignment at birth, NICU admission, interfacility transfer pathways, payment source, and county access-category comparisons) were restricted to births occurring during 2014–2024.

# Outcomes: neonatal mortality age windows

Age at death in days was calculated as death date minus birth date, and mortality indicators were created by flagging deaths within each age window. For descriptive analyses, we also constructed neonatal mortality (<28 days) and infant mortality (<365 days) indicators.

# Clinical risk strata: gestational age and birth weight

To characterize clinical risk, births were stratified by gestational age (GA) and birth weight (BW). GA and BW were analyzed both as categorical strata and as components of required level-of-care assignment. Stratified mortality rates (per 1,000 live births) were estimated with 95% confidence intervals.

# Required neonatal level of care and triage alignment

Triage alignment was operationalized by comparing the required level of care to the facility neonatal level at the relevant point in the care pathway:

- **_Birth triage:_** facility level was defined as the neonatal level of the delivery hospital.
- **_Transfer triage:_** facility level was defined as the neonatal level of the receiving hospital.

Care was classified as risk-appropriate when required and facility levels matched exactly, under triage when the facility level was below the required level, and over-triage when the facility level exceeded the required level. For transfer triage, receiving facilities were mapped to neonatal levels using the study facility registry; facilities without an identifiable NICU designation were manually labeled and were excluded from triage classification. Under-triage among transfers was rare; due to sparse counts, under-triaged transfer cases were excluded from inferential comparisons of transfer-related mortality.

# County maternity care access categories

Maternal home counties were categorized into four maternity care access strata (Full, Moderate, Low, Desert) based on standardized county-level access classifications.^4^ For longitudinal consistency, access categories were treated as stable across the study period.

# Obstetric unit closure analysis

We conducted pre–post analyses of obstetric unit closures with sufficient follow-up during the study period, including one urban Level II closure and two rural Level I closures. For each closure event, we defined a pre-closure period as all months preceding the closure month and a post-closure period as the closure month through December 2024. Within the maternal home county most directly affected, we computed pre- and post-closure mortality rates (per 1,000 births) and compared periods using two-sided Fisher’s exact tests. To contextualize county-specific changes, benchmark mortality rates were calculated for the entire state and for the closure county’s access-category peer group over the same pre/post windows.

# Prenatal care analysis

We evaluated prenatal care utilization in relation to early neonatal mortality (defined as death at 0–6 days via linked birth and death certificates) for the 2014–2024 cohort. Prenatal care exposure was operationalized in two ways: a binary indicator based on the birth-certificate prenatal field (prenatal = Y versus prenatal = N/U) and a measure of visit intensity using the total recorded prenatal visits (birth_prenatal_vis_cnt). We first compared early neonatal mortality rates (per 1,000 live births, with 95% Wilson score confidence intervals) between the binary groups using *𝜒*^2^ tests of independence, reporting Cramér’s *𝑉* as an effect size. To assess dose-response dynamics, we compared mortality rates across categorical visit groups (0, 1–2, 3–4, 5–7, 8–10, and ≥11) using *𝜒*^2^ tests and conducted a complementary continuous analysis by modeling the mortality rate against visit count using a birth-volume-weighted linear trend. Finally, to separate prenatal care utilization from biological and geographic drivers, these analyses were repeated within strata of birth weight, gestational age, and county maternity care access level; within each stratum, differences in the number of prenatal visits between infants who experienced early neonatal death and those who survived were assessed using two-sided Mann–Whitney U tests.

# Statistical analysis

Mortality rates were reported per 1,000 live births with 95% confidence intervals using the Wilson score method. Population-level comparisons across county access categories and payment source were assessed using *𝜒*^2^ tests of independence on birth-level contingency tables, with effect sizes summarized using Cramér’s *𝑉*. When pairwise comparisons were performed, we used two-sided Fisher’s exact tests with Bonferroni correction for multiple comparisons (e.g., *𝛼* _=_ 0*.*05/6 = 0*.*0083 for comparisons across four county access categories). County-level analyses treated each county as a single observation using pooled rates (e.g., county-year or pooled 2014–2024 rates, depending on the analysis); within-group variability was summarized using medians and interquartile ranges (IQR), and differences across access categories or temporal stability across years were assessed using Kruskal–Wallis tests with Dunn’s post-hoc comparisons when the overall test was significant. For analyses comparing mortality across triage categories, we used *𝜒*^2^ tests on death versus survival contingency tables and reported Cramér’s *𝑉*; when sparse counts occurred, categories were collapsed for stable estimation. For pre–post obstetric unit closure analyses, differences in county-level mortality were evaluated using Fisher’s exact tests and contextualized using matched state and access-group benchmark rates over the same windows. All tests were two-sided with *𝛼* = 0*.*05.

# Matched Case-Control and Multivariable Analysis

The primary outcome for the multivariable analysis was early neonatal death. A matched case-control design was used to compare early neonatal deaths with surviving infants. Cases were matched to up to three controls by birth year, maternal county of residence, birthweight category, and gestational-age category. When exact matches were unavailable, relaxed matching criteria based on continuous birthweight, gestational age, and county-level maternity care access were used. Infants with a recorded death at any age were excluded from the control pool.

Conditional logistic regression was used for the primary inferential analysis to account for the matched design. Odds ratios and 95% confidence intervals were estimated for early neonatal death. When the standard model did not converge cleanly or showed evidence of quasi-separation, we used Firth-penalized conditional logistic regression, consistent with prior work on bias reduction in matched case-control conditional logistic regression and rare-events logistic regression. This was particularly relevant for congenital anomaly, which was present in 100 cases but only 2 controls across the matched sample, and for neonatal seizures, which were present in 8 cases and 2 controls.

Nested models were estimated to evaluate how associations changed with increasing adjustment. Model 1 included the primary exposure variables: under-triage, high travel distance to the delivery facility, prenatal visit category, and payment source. Model 2 added continuous gestational age and birthweight scaled per 100 g to account for residual biological differences within the matched gestational-age and birthweight categories. Model 3 further adjusted for clinical and demographic factors, including 5-minute Apgar categories, assisted ventilation, prolonged ventilation, congenital anomaly, seizures, multiple birth, infant sex, and maternal race.

Prenatal care was modeled using visit-count categories: 0 visits, 1–3 visits, 4–7 visits, and 8–10 visits, with 11 or more visits used as the reference group. In this model, birthweight was scaled per 100 g, and payment source was modeled using private insurance as the reference group, with Medicaid and self-pay combined as a single category.

# Supplementary Machine Learning Analysis

Extreme gradient boosting (XGBoost) was used as a supplementary, non-parametric prediction model to explore variable importance within the matched analytic sample. The binary outcome was early neonatal death, and the feature set included biological, clinical, prenatal care, access, payer, and demographic variables used in the regression workflow. The classifier was fit with 300 estimators, a maximum tree depth of 5, a learning rate of 0.05, and row and column subsampling rates of 0.8. Class imbalance was addressed using a weighting parameter based on the ratio of surviving controls to early neonatal deaths.

Predictive performance was evaluated using 5-fold cross-validation that preserved matched sets within folds and was summarized using the area under the receiver operating characteristic curve

and the Brier score. After cross-validation, a final XGBoost model was fit on the full matched analytic sample to estimate SHapley Additive exPlanations (SHAP) values for global feature-importance ranking. Mean absolute SHAP values were used to rank predictors, and SHAP beeswarm plots were used to visualize both effect magnitude and direction. Because the machine learning analysis was exploratory and prediction-oriented, SHAP rankings were interpreted as predictive contributions rather than adjusted causal effects.

# Supplementary Results

### XGBoost

The XGBoost model was used to explore which variables were most useful for prediction, not to estimate causal effects. Therefore, SHAP values were interpreted as showing how much each variable contributed to prediction, rather than as adjusted odds ratios or causal associations. In 5-fold cross-validation, the model showed good discrimination for early neonatal mortality, with a mean AUC of 0.901 (SD 0.013), and a mean Brier score of 0.104 (SD 0.006).

The strongest predictors were direct biological and clinical severity indicators. The 5-minute Apgar score was the most important feature by mean absolute SHAP value (mean |SHAP| = 1.32), followed by birth weight (mean |SHAP| = 0.65), gestational age (mean |SHAP| = 0.54), prenatal visit count (mean |SHAP| = 0.31), severe 5-minute Apgar depression (mean |SHAP| = 0.25), maternal age (mean |SHAP| = 0.23), and congenital anomaly (mean |SHAP| = 0.15). These results indicate that early neonatal mortality prediction was driven primarily by infant condition at birth, prematurity, fetal growth, and prenatal care patterns.

Access and sociodemographic variables also contributed to prediction, although their relative importance was lower than the direct biological and clinical markers. Maternal race, high travel distance to the delivery facility, payment source, multiple birth, ventilation indicators, under-triage, and any prenatal care appeared among the top 20 features. High travel distance had a mean |SHAP| of 0.10, while under-triage had a mean |SHAP| of 0.03. Overall, the SHAP analysis was consistent with the conditional logistic regression results in showing that biological severity dominated mortality prediction, while prenatal care, travel distance, payer, and triage variables added additional predictive information (Supplementary Figure S2).

# Section 2: Supplementary Tables, and Figures

## 1. Early Neonatal Mortality by Birth Facility Appropriateness Stratified by Birth Weight and Gestational Age

**Table S1. Early Neonatal Mortality by Birth Weight and Birth Facility Appropriateness**

| **Stratum** | **Triage Category** | **Deaths** | **Births** | **Rate per 1,000** | **95% CI** |
| --- | --- | --- | --- | --- | --- |
| ELBW (<1000 g) | Risk-Appropriate | 497 | 2,143 | 231.918 | [214.538, 250.257] |
| ELBW (<1000 g) | Over-Triage | 0 | 0 | NaN | Na |
| ELBW (<1000 g) | Under-Triage | 297 | 666 | 445.946 | [408.612, 483.9] |
| VLBW (1000–1499 g) | Risk-Appropriate | 87 | 2,438 | 35.685 | [29.022, 43.809] |
| VLBW (1000–1499 g) | Over-Triage | 0 | 0 | NaN | Na |
| VLBW (1000–1499 g) | Under-Triage | 44 | 565 | 77.876 | [58.521, 102.932] |
| LBW (1500–2499 g) | Risk-Appropriate | 94 | 7,289 | 12.896 | [10.551, 15.755] |
| LBW (1500–2499 g) | Over-Triage | 48 | 20,406 | 2.352 | [1.775, 3.117] |
| LBW (1500–2499 g) | Under-Triage | 60 | 1,822 | 32.931 | [25.669, 42.158] |
| Normal (2500–3999 g) | Risk-Appropriate | 74 | 56,781 | 1.303 | [1.038, 1.636] |
| Normal (2500–3999 g) | Over-Triage | 64 | 262,227 | 0.244 | [0.191, 0.312] |
| Normal (2500–3999 g) | Under-Triage | 56 | 4,651 | 12.04 | [9.284, 15.602] |
| Macrosomia (≥4000 g) | Risk-Appropriate | 11 | 3,932 | 2.798 | [1.563, 5.003] |
| Macrosomia (≥4000 g) | Over-Triage | 4 | 21,151 | 0.189 | [0.074, 0.486] |
| Macrosomia (≥4000 g) | Under-Triage | 8 | 455 | 17.582 | [8.936, 34.307] |

**Table S2. Early Neonatal Mortality by Gestational Age and Birth Facility Appropriateness**

| **Stratum** | **Triage Category** | **Deaths** | **Births** | **Rate per 1,000** | **95% CI** |
| --- | --- | --- | --- | --- | --- |
| Extremely Preterm (<28 wk) | Risk-Appropriate | 506 | 2,098 | 241.18 | [223.36, 259.95] |
| Extremely Preterm (<28 wk) | Over-Triage | 0 | 0 | NaN | [NaN, NaN] |
| Extremely Preterm (<28 wk) | Under-Triage | 294 | 696 | 422.41 | [386.24, 459.44] |
| Very Preterm (28–31 wk) | Risk-Appropriate | 93 | 3,134 | 29.68 | [24.29, 36.22] |
| Very Preterm (28–31 wk) | Over-Triage | 0 | 0 | NaN | [NaN, NaN] |
| Very Preterm (28–31 wk) | Under-Triage | 30 | 708 | 42.37 | [29.84, 59.85] |
| Moderate Preterm (32–36 wk) | Risk-Appropriate | 93 | 8,189 | 11.36 | [9.28, 13.89] |
| Moderate Preterm (32–36 wk) | Over-Triage | 52 | 26,576 | 1.96 | [1.49, 2.57] |
| Moderate Preterm (32–36 wk) | Under-Triage | 60 | 2,315 | 25.92 | [20.19, 33.22] |
| Term (37–41 wk) | Risk-Appropriate | 85 | 58,982 | 1.44 | [1.17, 1.78] |
| Term (37–41 wk) | Over-Triage | 62 | 276,621 | 0.22 | [0.18, 0.29] |
| Term (37–41 wk) | Under-Triage | 78 | 4,432 | 17.6 | [14.13, 21.91] |
| Post-Term (≥42 wk) | Risk-Appropriate | 0 | 106 | 0 | [0.00, 34.97] |
| Post-Term (≥42 wk) | Over-Triage | 1 | 473 | 2.11 | [0.37, 11.88] |
| Post-Term (≥42 wk) | Under-Triage | 2 | 11 | 181.82 | [51.37, 476.98] |

**Note:** Rates are reported per 1,000 live births unless otherwise noted. Confidence intervals are Wilson 95% confidence intervals for proportions/rates. Statistical tests are reported as calculated in the supplemental analyses.

## 1. Early Neonatal Mortality by Transfer Status

Figure S1. Early neonatal mortality by transfer status.

## 2. XGBoost SHAP analysis for early neonatal mortality.

Figure S2: Supplementary XGBoost SHAP analysis for early neonatal mortality. Panel A ranks the top 20 predictors by mean absolute SHAP value, and Panel B shows the SHAP beeswarm plot illustrating effect magnitude and direction.

## 3. Early Neonatal Mortality by Maternal Race

Figure S3. Early neonatal mortality by maternal race.

## 4. Prenatal Care Access by Maternal Race

Figure S4. Any prenatal care received by maternal race.

## 5. Prenatal Care Utilization by Maternal Race

Figure S5. Prenatal visit intensity by maternal race among births with any prenatal care.
